# Advances in AI and machine learning for predictive medicine

**DOI:** 10.1038/s10038-024-01231-y

**Published:** 2024-02-29

**Authors:** Alok Sharma, Artem Lysenko, Shangru Jia, Keith A. Boroevich, Tatsuhiko Tsunoda

**Affiliations:** 1 Laboratory for Medical Science Mathematics, Department of Biological Sciences, School of Science, The University of Tokyo, Tokyo, Japan; 2 Laboratory for Medical Science Mathematics, RIKEN Center for Integrative Medical Sciences, Yokohama, Japan; 3 Institute for Integrated and Intelligent Systems, Griffith University, Queensland, Australia; 4 Laboratory for Medical Science Mathematics, Department of Computational Biology and Medical Sciences, Graduate School of Frontier Sciences, The University of Tokyo, Tokyo, Japan

**Keywords:** Data mining, Computational biology and bioinformatics

## Abstract

The field of omics, driven by advances in high-throughput sequencing, faces a data explosion. This abundance of data offers unprecedented opportunities for predictive modeling in precision medicine, but also presents formidable challenges in data analysis and interpretation. Traditional machine learning (ML) techniques have been partly successful in generating predictive models for omics analysis but exhibit limitations in handling potential relationships within the data for more accurate prediction. This review explores a revolutionary shift in predictive modeling through the application of deep learning (DL), specifically convolutional neural networks (CNNs). Using transformation methods such as DeepInsight, omics data with independent variables in tabular (table-like, including vector) form can be turned into image-like representations, enabling CNNs to capture latent features effectively. This approach not only enhances predictive power but also leverages transfer learning, reducing computational time, and improving performance. However, integrating CNNs in predictive omics data analysis is not without challenges, including issues related to model interpretability, data heterogeneity, and data size. Addressing these challenges requires a multidisciplinary approach, involving collaborations between ML experts, bioinformatics researchers, biologists, and medical doctors. This review illuminates these complexities and charts a course for future research to unlock the full predictive potential of CNNs in omics data analysis and related fields.

## Introduction

Recent advances in genomics have bolstereed our capacity to delve deeper into the complex etiologies of various diseases. Among them, Genome-Wide Association Studies (GWAS) are one example of approaches driving these developments and have greatly improved our understanding of genetic factors that underpin many important complex traits and diseases. However, this research still faces many limitations that demand innovative solutions to unlock its full potential. Many of these problems arise from the insufficient ability of current modeling methods to capture the full degree of underlying biological complexity. The number of genetic variants is vast, and their individual effects are often small and context-specific. Fully mapping out the mechanisms that translate genetic variation into phenotypes requires fine integration of genetic analysis with other types of knowledge and ‘omics domains. By their nature, these aspirations closely align with problems currently being encountered and solved in other data-intensive domains, both in other areas of biology and more widely. In this review we will outline promising synergies and solutions from recent advances in DL analysis and explain their potential applications in biomedical research.

Extensive datasets offer insights into the molecular mechanisms behind various diseases and create novel opportunities for predictive modeling in precision medicine, particularly in the realm of drug efficacy prediction. However, complexity and volume of omics data pose significant challenges in extracting meaningful information and deciphering essential biological context. Within high-throughput genomics, similar to other omics domains, technological advances have brought with them both the typical challenges associated with overabundance of data and also unique problems – like those arising from the methodological specifics of GWAS. Meaningful interpretation of such data is complicated, as most contemporary profiling strategies capture an outwardly extensive yet functionally narrow view of the entire biological system. Some key challenges currently affecting genomics research within the context of these overarching themes are briefly outlined below.

One often-encountered criticism of the GWAS approach is its focus on common genetic variants with modest effects that often do not explain a significant part of the genetic contribution to complex traits [1]. This issue arises due to the presence of rare variants with very large contributions, complex epistatic effects, and interactions between genes and the environment. Even once a GWAS study has led to identification of potentially relevant variants, it is still essential to determine their functional consequences and causality, which can often be complicated and require additional targeted experiments [2]. The task of distinguishing causal variants from other markers in linkage disequilibrium blocks and understanding their mechanistic effects on observable phenotypes require some form of additional information. This type of research will therefore greatly benefit from reliable, automated integration of functional genomics [3], epigenomics [4], and transcriptomics [5].

Attempting to explain ever more complex natural processes inevitably requires comparably complex models. This necessitates the use of advanced algorithms to automate model construction and the fitting process, which results in ‘black-box’ models that, although very accurate, usually lack human interpretability. Increasing complexity can ultimately limit the usefulness of these methods for biological discovery (as it can obscure hints about potential mechanisms) and undermine trust in such tools for clinical applications, one example being polygenic risk scores for complex diseases [6]. Additionally, many genetic studies have to deal with suboptimally small numbers of samples – especially in the case of rare diseases, variants with subtle effects or those confined to specific population subgroups. Artificial intelligence (AI) and ML techniques [7, 8] offer multiple new strategies to address these limitations and, most importantly, improve our ability to integrate knowledge and data across multiple layers of biological organization.

ML algorithms encompass a broad range of computational methods that enable computers to construct predictive models leading to actionable insights. In medical genomics, ML techniques have found diverse applications, including but not limited to, predictive disease classification, biomarker discovery, drug response prediction, and identification of disease-causing genetic variants. Classical ML techniques, such as Random Forest [9], Support Vector Machines [10], and logistic regression [11], have been instrumental in omic data analysis. These methods excel at handling high-dimensional data and have demonstrated success in various genomics research endeavors. For a comprehensive survey on the typical applications of standard ML techniques in biomedical research, readers are referred to existing review articles [12–17]. However, as the dimensions and layers of data grow, so do the limitations of these classical methods. One significant drawback is their insensitivity to the relationships behind data, e.g. genes often crucial in omics, because they are mostly from “tabular” data, where variables are represented independently with each other. Seizing the potential relationships of genes or elements can offer a wealth of information as we describe in detail below.

The emergence of DL has revolutionized the field of AI and transformed the landscape of data analysis. This ability of DL to automatically learn hierarchical representations from raw data has proven invaluable for predictive modeling, capturing intricate dependencies within datasets, even when dealing with noisy and high-dimensional data. One particularly important development in the area of DL is the creation of new methods to deal with small sample sizes, a recurring concern in genetic profiling of small populations and rare diseases. While the ideal application of DL aims to facilitate comprehensive representation learning of the underlying structures within omics data, practical challenges emerge when too few samples are available. In certain applications this can even hinder adequate representation learning and complicate the direct processing of omics data within DL frameworks. This important limitation can be mitigated using a technique called transfer learning. Unlike classical statistical models, neural networks are fitted sequentially meaning that they can be continuously updated with new data. This opens a possibility of ‘pre-training’ a model on some large, but weakly or partially relevant dataset and then finalizing the training on a more valuable but smaller one. For patterns that persist between the two datasets there will be a benefit from access to all of the combined observations, whereas irrelevant patterns will simply be overwritten with new data. Transfer learning can facilitate the reuse of knowledge from larger datasets to substantially improve accuracy in smaller cohorts. In principle, pre-trained models initialized on large-scale genetic data can be re-specialized for new tasks, reducing the need for data collection while also improving performance. The potential and limitations of this strategy are discussed in detail in this review and its benefits are demonstrated with several examples [18–24].

DL models offer a wide range of additional analysis capabilities that can improve many kinds of high-throughput biomedical analysis. Firstly, artificial neural networks can easily identify large numbers of interactions and model non-linear effects while also offering very effective regularization to mitigate the risk of overfitting [25]. Secondly, modern neural networks can utilize very large input sizes, incorporate methods for imputation of missing values [26] and accommodate very diverse types of structured information. All of these capabilities offer additional ways of increasing power to detect rare SNPs, epistasis, and more accurately model the full range of possible association patterns. DL models can jointly analyze and integrate heterogeneous data sources, allowing for a more comprehensive view of genetic contributions and some recently introduced methods offer ways to integrate different omics data types within the same model and perform inference across them simultaneously. Of particular note is the DeepInsight family of approaches [18–21], where several studies have demonstrated successful cross-omics analysis that included cancer somatic variation as one of the input types. As this type of data is akin to germline variation, potentially similar strategies can be used in the future for enhancing functional and causal annotation of SNPs.

Lastly, increasing use of AI across all areas of life has brought to the fore the need for such systems to become more transparent and interpretable. This problem is a focus of increasing research interest and explainable AI (XAI) is now emerging as its own sub-discipline within AI research. As the vast majority of this work was done with DL-based models, most of these methods can be readily used with most typical architectures of neural networks. Techniques like attention and gradient-based attribution can in principle help to understand the contribution of individual biological factors to disease risk and drug response, making the results more interpretable for biologists and clinicians. To demonstrate these potential benefits, this review will use DeepFeature method as an illustrative example, which implements a gradient-based attribution approach to discover potential mechanisms involved in cancer drug efficacy [20, 21].

Here we focus more on biologically inspired CNNs [27], which are one of the fundamental architectures widely used in the computer vision domain, and their adoption to it has led to unprecedented improvements in performance. Two-dimensional (2D) CNNs are extensively used tools, particularly for image data analysis, as they extract spatial features hierarchically, starting from raw image data, through edge-detection etc., and finally for object prediction. While 2D CNNs have traditionally thrived in image analysis, recent interest has arisen in their application to omics data analysis. Researchers have contemplated the prospect of harnessing the potency of 2D CNNs for tabular or omics data analysis, necessitating the revelation of latent (we sometimes call “spatial”) information inherent among genes (or elements) within a sample (or feature vector) [28–31]. Zhou et al. [32] underscored the significance of DL including CNN in predictive tasks like determining the sequence specificity of DNA- and RNA- binding proteins and pinpointing cis-regulatory regions, among other applications. Notably CNNs and recurrent neural networks have become the architectures of choice for modeling these regulatory elements with sequence patterns, illustrating the wide-ranging utility of DL in genomics. Talukder et al. further explore the intricacies of deep neural network (DNN) interpretation methods, particularly their applications in genomics and epigenomics [33]. This breadth of application also extends to synthetic biology, emphasizing its promise in plant and animal breeding [34]. Nonetheless, existing reviews have not extensively addressed how to effectively handle tabular data like omics data without explicit patterns by converting them to adequate representations for CNNs.

With the emergence of converter techniques like DeepInsight [18], a groundbreaking development has transpired: the conversion of tabular data, such as omics data, into image-like representations. This transformative conversion now empowers the effective harnessing of CNNs for analysis. DeepInsight, a pioneering technique, revolutionizes data preprocessing by instilling latent information among genes or elements within a feature vector. This reimagining of data arranges elements sharing similar characteristics into proximal neighbors, while distant elements remain distinct. This spatial context generates a rich environment for CNNs to operate not only feasibly but also insightfully. Unlike traditional ML techniques, which independently handle variables and sometimes pick representative ones, this new technique gathers similar variables close together and treats them as a group, which reflects the structure behind the omics data.

To clarify further, when biological data is transformed into an image format, the latent relationships between biological entities, such as genes, are encoded as spatial proximities within the image. Subsequently, using a CNN with these images allows for a substantial reduction in the number of model parameters. This reduction is achieved through the architectural design of convolutional layers, which are adept at identifying opportunities for parameter sharing among appropriate inputs, specifically in cases characterized by partial linear or even non-linear correlations among features. Given the prevalence of such correlations in biological data, the resulting models usually have better generalization capabilities, while preserving the neural networks’ innate ability to discover and model more complex features if and when needed. Moreover, these images facilitate the interpretation of results by explicitly showing the potential relationship between the biological entities found to be important by the model, as will be explained in detail further on.

One additional notable advantage is the capability of utilizing transfer learning, negating the necessity for network creation from scratch. This attribute allows for all-encompassing learning across a diverse spectrum of omic data, unlocking novel avenues for comprehensive analysis. Through transfer learning, models can be initialized with weights from a pre-trained model [35], typically developed using extensive and diverse image datasets such as ImageNet. These pre-trained models have already learned essential patterns from millions of natural images, hierarchically capturing universal features that are surprisingly effective when repurposed for distinct tasks, even in seemingly unrelated domains like genomics. This approach allows researchers to capitalize on the foundational knowledge embedded in these pre-trained models, drastically reducing the computational effort and time required for training, and often enhancing performance.

Utilizing transfer learning with pre-trained models offers a unique advantage for omic data analysis [18, 19]. Genomic datasets, unlike publicly available image datasets, are often limited in size. Leveraging the pattern learned by models from vast image datasets through transfer learning can provide a robust foundation, enabling researchers to fine-tune these models for the specifics of omics data, without the need for large training sets. Additionally, transfer learning allows for the extraction of intricate and nuanced patterns from omics data that might be overlooked or unattainable when starting the model training from scratch. The prowess of transfer learning using CNNs is vividly showcased in various applications beyond just image processing, demonstrating their potential to revolutionize data analysis across fields [18, 22, 36, 37].

**Fig. 1:**
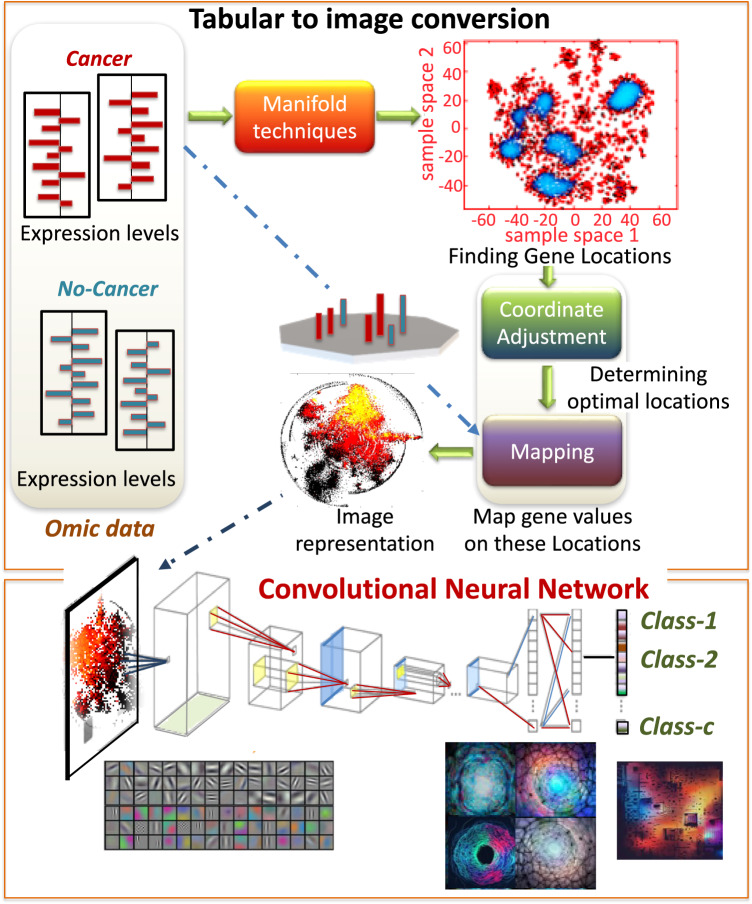
Tabular-to-image conversion with DeepInsight. This composite figure illustrates the transformation of tabular omic data into an image format for analysis using convolutional neural networks (CNNs). Comparative expression levels of genes in cancer versus non-cancer samples are input as tabular omic data. A manifold technique is applied to find optimal gene locations in a two-dimensional sample space, depicted by a scatter plot. The optimal gene locations are then adjusted for coordinate mapping and overlaid onto a grid to create an image representation where each point corresponds to a gene expression value. The resulting image is processed through a CNN, where the architecture is trained to classify the samples into categories, shown here as Class-1, Class-2, and Class-c, based on the learned patterns from the image data

The adaptability of DeepInsight is evident through its applications across various domains, including its pivotal role in shaping the winning model ('Hungry for gold') of the Kaggle.com competition [19, 20, 22, 23, 38–46]. For an in-depth exploration, those curious can delve into a comprehensive compendium of image conversion methods and their applications, as elucidated by Ye and Wang [47]. This transformative progression in data transformation and analysis signifies a momentous stride forward not only for unraveling the intricate nuances ingrained within tabular data but also for enhancing its predictive modeling capabilities. The schematic representation of the tabular-to-image converter process for using CNN is illustrated in Fig. 1.

## Challenges in advancing CNN applications in omics analysis

While the amalgamation of tabular-to-image conversion with CNNs for omics analysis has propelled significant advancements, a panorama of challenges and issues still beckon resolution. These include:

1. Interpretability: DL models, including CNNs, are often considered as “black box” due to their complex architectures. The ability to comprehend the specific genes or elements influencing a model’s decisions is pivotal in elucidating biological mechanisms, such as pathways. Although techniques like DeepFeature [20], which leverages class activation maps (CAMs) [48], have been introduced, the challenge remains open, necessitating the development of models to interpret learned features for deeper insights.
2. Data heterogeneity: Omics data is intrinsically heterogeneous, spanning diverse biological information types such as gene expression, methylation, and mutation. Adapting various omics data types while preserving each latent structure poses a challenge.
3. Data scaling and size: DL models, including CNNs, demand substantial data quantities for effective generalization. However, omics datasets, especially those associated with rare disease or specific conditions, may have limited sample sizes. Overcoming the constraints of small-scale datasets and ensuring the model’s robustness are vital considerations.
4. Overfitting: Traditional ML methods, particularly when dealing with high-dimensional omics data, have been known to be susceptible to overfitting. This led to an understanding that model complexity should be carefully managed to prevent such overfitting. However, recent theoretical advancements are challenging this view, particularly in the realm of DL. Specifically, DL algorithms possess intrinsic regularization features within their back propagation learning process. Intriguingly, these features can actually reduce the risk of overfitting as the network scales - contrary to what one might expect, adding more nodes or layers can make the model more robust. This upends our traditional understanding from classical statistics and ML, where increased model complexity usually exacerbates overfitting. Therefore, while the importance of balance model complexity, capacity, and data still holds, these new insights suggest that the considerations for achieving this balance in the context of DNNs may be fundamentally different.
5. Hyperparameter tuning: DL models encompass multiple hyperparameters influencing their performance. Identifying the optimal set of hyperparameters for specific omics datasets can be time-intensive, demanding expertize. Bayesian optimization techniques offer avenues for exploring optimal hyperparameters.
6. Computational resources: Training DL models, especially CNNs, can strain computational resources. For researchers with limited resources, optimizing the training process and exploring techniques like transfer learning becomes crucial.
7. Biological relevance: While models convert omics data into image-like representations, preserving the biological relevance of these representations remains paramount. Validating the meaningfulness of transformed data in terms of capturing underlying biological mechanisms stands as a challenge.
8. Generalizability: Ensuring the generalizability of a model across varying experimental conditions, platforms, and biological contexts demands attention. While recent efforts have integrated single-cell data from different platforms for cell identification in the context of tabular-to-image conversion with CNN application [21], further research in this direction is warranted.
9. Integration with domain knowledge: Infusing domain-specific knowledge into the model training process enhances interpretability and result relevance. Developing methods to seamlessly integrate prior biological knowledge with CNN-based analysis holds promise.
10. Benchmarking and comparison: Rigorous benchmarking against established methods and cross-dataset comparisons are vital for evaluating the true potential of a model.

**Fig. 2:**
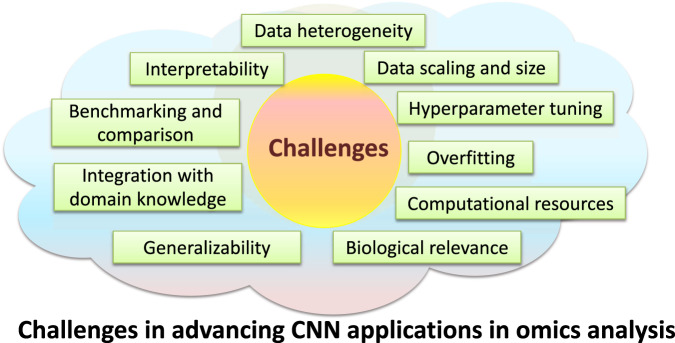
Overview of challenges in CNN applications for omics data. The figure outlines the ten main challenges in the use of convolutional neural networks (CNNs) for omics analysis, including interpretability, data diversity, model overfitting, computational demands, and the necessity for robust benchmarking to ensure biological validity and generalizability, which need to be addressed to advance the field

A summary of these issues is depicted in Fig. 2. Addressing these multifaceted challenges necessitates interdisciplinary collaboration between ML experts, bioinformatics researchers, and biologists. This collaboration is pivotal in advancing the integration of tabular-to-image converter models with CNNs, thus propelling the horizons of omics data analysis and interpretation.

## DeepInsight and DeepFeature: a new quirk in omic data analysis

The application of ML to genomics has primarily been within the realm of tabular (table-like, including vector) data analysis. Yet, with the advent of new methodologies, we are now able to bridge the gap between tabular and image data analysis, enhancing the extraction of meaningful insights from omic datasets.

### DeepInsight: transforming tabular data to image-like form and use of pre-trained CNN

**Fig. 3:**
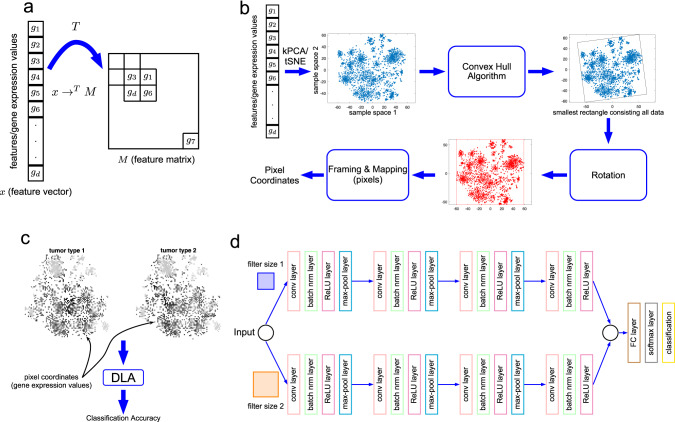
DeepInsight pipeline and network. **a** Transformation of the feature vector into a feature matrix. **b** Pipeline of converting a feature vector into image pixels. **c** Visualization of two tumor types using DeepInsight’s image transformation method. Differences between the tumor types are observable at various points. These transformed images are subsequently fed into a deep learning architecture (DLA). **d** The CNN architecture used in the original DeepInsight paper. This architecture consists of two parallel CNN architectures where each consists of four convolutional layers. Parameters are tuned using Bayesian Optimization technique. However, one can use various pre-trained CNN architectures. (From DeepInsight paper [18] under creative commons license.)

At the forefront of this transformation approach is DeepInsight [18], a methodology designed to convert tabular data (including omic data) into image-like representations reflecting the latent structure behind the data. Figure 3 provides an illustration of the DeepInsight pipeline. Briefly, a feature vector, $x$, containing gene expressions or elements is transformed to a feature matrix $M$ through a transformation $T$. The placement of individual features within this matrix depends on their similarities, as shown in Fig. 3a. Once locations for the features are determined, their expression or element values are mapped onto these positions. The transformation process consists of several key steps, as shown in Fig. 3b:

1. Placing the genes or elements on the Cartesian coordinates using manifold methods such as t-SNE, UMAP, or kernel PCA.
2. Utilizing the convex hull algorithm to find the smallest rectangle that encapsulates the feature spread, followed by rotation to align with the horizontal and vertical axes.
3. Converting the Cartesian coordinates to a pixel framework.
4. Mapping the values of elements or gene expression onto their corresponding positions within this pixel framework.

In this conversion process, similarity between genes or other factors of interest is represented by relative closeness of their spatial positions. This encoding ensures that elements with similar characteristics are positioned proximal to each other, while those that are dissimilar are distant. This transformation yields an image representation equivalent to the original feature vector, as shown in Fig. 3c. These generated images serve as input for CNNs in subsequent predictive modeling, as depicted in Fig. 3d. Further improvements expanded on this paradigm, such as adding blurring technique to DeepInsight [49], fusion with Gabor filtering [50], with autoencoders [24], aligning features into different layers (to avoid averaging) [38, 46], transforming to fixed-pixel framework [45], and multifacet representation model [51]. The original DeepInsight has been further refined and was first adapted for integration with Vision Transformers (ViT) by Gokhale and team, making a significant leap forward in this field [24].

The resulting image-like representations are ideally suited for analysis by CNNs. Furthermore, as mentioned above, DeepInsight facilitates the use of pre-trained CNN models, which have historically excelled in image analysis. The benefit of such an approach is twofold: it not only capitalizes on the robust capabilities of pre-existing CNN architectures but also offers accelerated insights by eliminating the need to train models from scratch.

To evaluate the performance of DeepInsight, several scenarios were examined, as detailed in [18], including cancer-type prediction, in which the method delivered an improved performance relative to several other ML methods. In a subsequent study, DeepInsight-3D [19] was compared to multiple neural network architectures (feed forward, autoencoder, ANNF), optimized random forest pipeline (AutoBorutaRF), support vector machine-based classifier, and three recent drug response prediction pipelines (Gelleher et al. model, MOLI and Super.FELT). DeepInight-3D showed a 7–29% improvement in performance, as measured by model AUC-ROC, across all these methods.

### DeepFeature: extracting features with CAM

**Fig. 4:**
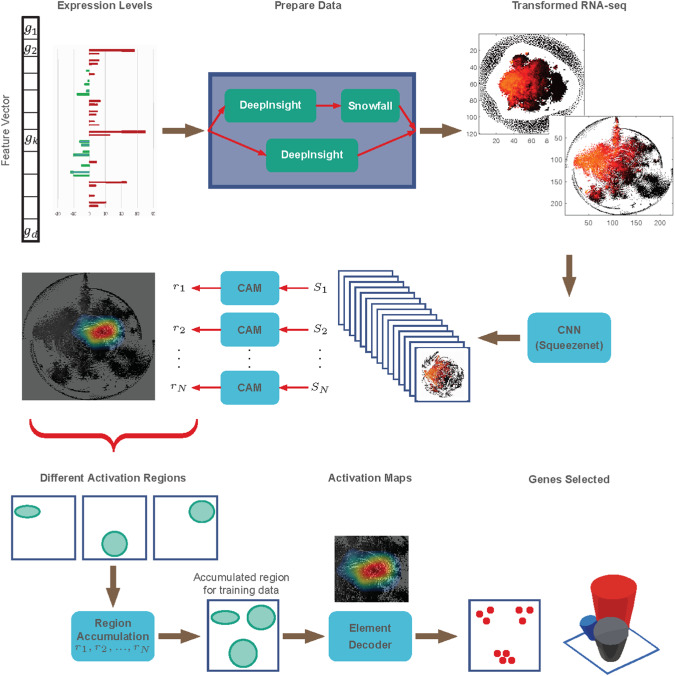
Overview of the DeepFeature procedure for feature selection using CNN. (From DeepFeature paper [20] under creative commons license.)

DeepFeature [20] complements the analytical capabilities introduced by DeepInsight. While DeepInsight focuses on data transformation, DeepFeature targets the interpretability challenge, particularly in the context of DL models. Utilizing CAMs [48], DeepFeature extracts and highlights the pivotal features that influence a model’s decisions, e.g. prediction. In genomics, this translates to identifying key genes or elements that are crucial in determining specific phenotypic outcomes or disease manifestation. Figure 4 illustrates the DeepFeature methodology pipeline. An input vector of tabular data is presented in the upper left, leading to the selected features or genes displayed in the lower right.

The biological implications are profound, especially when analyzing cancer types as described above. By transforming omic data of various cancer samples into image-like formats, researchers can use CNNs to discern patterns and differences that might be elusive in traditional tabular analysis. The feature extraction capabilities of DeepFeature further enrich this analysis. By highlighting genes or elements of significance inside the CNNs through visualization techniques like CAM, researchers can derive deeper insights into the molecular mechanisms driving different cancer types. When applied to the cancer-type identification task described above, it could extract many more known cancer related genes/pathways than traditional statistical modelings or ML methods like penalized logistic regression, and also could discover new pathways for the classification of different types of cancer. Such insights have the potential to elucidate pathways that are activated or suppressed in specific cancer forms, paving the way for targeted therapeutic strategies and personalized medicine.

In summary, the innovative transformation of omic data to images offers a paradigm shift in omic analysis. Through advanced data transformation and feature extraction, these methodologies provide a more refined lens to probe the complex world of genomics, particularly in understanding the intricacies of various diseases like cancer. That is, these methodologies can conduct “scientific discovery” from “big data”.

## DeepInsight-3D

To address the issue of heterogeneous data modality across multi-omics as mentioned above, we have conceptualized and developed DeepInsight-3D [19], an extension of the original DeepInsight, tailored specifically for multi-omic analyses.

### Extension of DeepInsight for multi-omic analysis

**Fig. 5:**
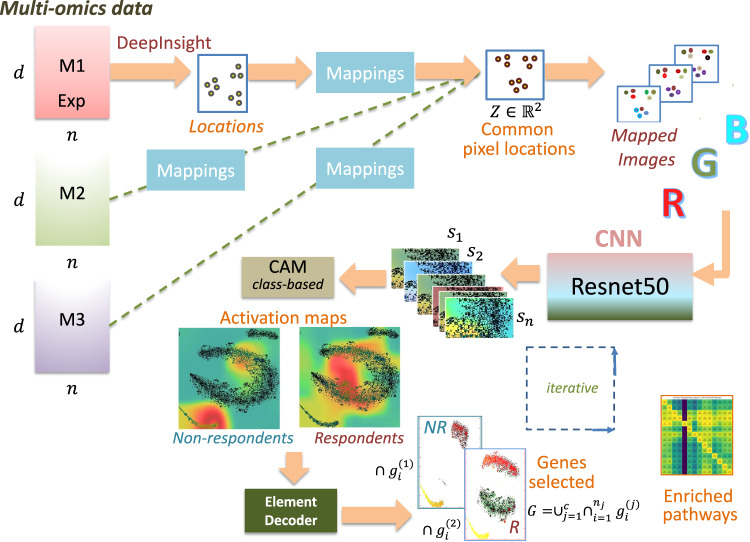
Overview of the DeepInsight-3D model. Starting from the left, multi-omics layers undergo processing via the DeepInsight methodology to identify common pixel locations. Upon mapping omics data, the corresponding images are constructed and then fed into a CNN architecture. Subsequently, CAM is employed to determine activation regions, while the element decoder pinpoints a subset of genes. (From DeepInsight-3D paper [19] under creative commons license.)

DeepInsight, originally designed to transform tabular omic data into image-like representations, paved the way for harnessing the computational prowess of pre-trained CNNs for genomics. DeepInsight-3D takes this a notch higher. By adapting to multi-omic data, it integrates information across different omic types into a unified three-dimensional (3D) space. This 3D representation captures the synergistic interactions among different omic data types, facilitating a holistic understanding and offering a richer context for analysis. Figure 5 provides a graphical depiction of the DeepInsight-3D model. The multi-omics data is displayed on the left-hand side, culminating in the selection of genes after the Element Decoder block (in the lower middle of Fig. 5).

### Application to anti-cancer drug response prediction

Recent advancements in the fields of AI, especially ML and DL, have shown remarkable potential in predictive modeling for drug response in various diseases, including cancer. The applicability of these computational techniques ranges from characterizing molecules numerically for ML algorithms [52] to evaluating the generalizability of drug response models [53]. Complex DL methods, in particular, have been employed to predict drug responses in cancer cell lines, although challenges like overfitting to limited data sets still exist [54]. These computational methods offer avenues for more accurate, individualized treatment options, providing a significant impact on precision medicine and healthcare [55].

One of the applications of DeepInsight-3D is in the realm of oncology, specifically for anti-cancer drug response prediction. Although limited drug response data cause the model’s stability issue, this concept is a step forward in handling multi-modality of data. By representing multi-omic data in 3D, DeepInsight-3D can capture the complex interactions of genes. When combined with patient-specific data, this tool offers predictions on how a tumor might respond to a specific anti-cancer drug. This kind of prediction holds the potential to revolutionize medicine by guiding therapeutic decisions based on individual patient profiles.

In the DeepInsight-3D paper, multi-omics data of gene mutations, gene expression, and copy number alterations were input to make the prediction model of drug efficacy. The mapping of data points was determined from the expression data by DeepInsight and positioned the mutations and copy number alterations to the gene positions, with different colors according to their levels. Cancer Cell Line Encyclopedia (CCLE) and Genomics of Drug Sensitivity in Cancer (GDSC) cell lines accompanied with drug efficacy, and The Cancer Genome Atlas (TCGA) and patient-derived xenografts (PDX) datasets were used for learning and testing the CNN, respectively. As a result, it showed 72% accuracy, which outperformed other deep-learning based methods by more than 7%. This clearly proved the power of the transforming scheme of DeepInsight.

### Feature selection with DeepFeature and pathway analysis

Representing and predicting aren’t enough; understanding the why and how behind these predictions is paramount, especially in a clinical setting. DeepFeature used in tandem with DeepInsight-3D, extracts crucial features using CAMs, highlighting specific regions in the 3D representation that significantly influence predictions. This feature selection is crucial not just for model interpretability but also for guiding subsequent biological investigations.

Furthering our understanding, pathway analysis, post DeepFeature extraction, deciphers the biological significance of these influential features. For instance, in the context of anti-cancer drug response, identifying pathways associated with drug resistance or sensitivity can shed light on potential molecular targets and therapeutic strategies. DeepInsight-3D has identified many pathways known to be involved in multiple drug responses: STAT3, PI3K/AKT, JAK/STAT, Rho GTPase, protein degradation and recycling, extracellular structure and cell adhesion, and could find new pathways: tryptophan metabolism X, and clathrin-dependent endocytosis. Having said that, stability is an issue with DeepInsight-3D and the limited scale of data can prompt incorrect predictions. Therefore, such considerations should be given when estimating the model. Nevertheless, the successful integration of multi-omics through DeepInsight-3D, combined with the feature extraction capabilities of DeepFeature, holds promise for myriad applications, from drug development to personalized therapy.

## scDeepInsight

### Unraveling the cellular landscape with enhanced precision - application to scRNA-seq data for cell-type identification

**Fig. 6:**
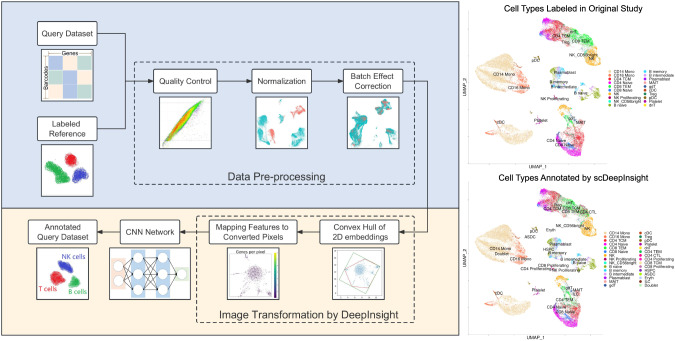
On the left, scDeepInsight’s workflow progresses from inputting the molecular identifier to generating cell annotations. After processing a reference dataset and query data, they are translated into 2D images, which are used for training the CNN model. Notably, only the reference data trains the model, while the query set is classified. For future query sets, the existing model can be directly applied without new training. On the right, a UMAP visualization of a query dataset shows labeled cell types from the initial study, contrasted with a UMAP colored by scDeepInsight’s predictions. (From Jia et al. [21] under creative commons license.)

Single-cell RNA sequencing (scRNA-seq) has opened new frontiers in understanding cellular heterogeneity, revealing insights that are often obscured in bulk RNA sequencing. The challenge, however, lies in the processing and interpretation of the high-dimensional data produced. Inputting a gene expression profile for a single cell from scRNA-seq, the cell type annotation task identifies the cell type from which the profile came from. Traditional cell type annotation methods rely on manual labeling of the unsupervised clustering results. This process requires the analysis of the expression results of specific marker genes. However, the available marker genes are limited and overlapping, especially for similar cell subtypes. The selection and subjective interpretation of marker gene lists also neglect the complex interrelationships among genes, further impeding accurate annotation. This is where scDeepInsight [21] comes into play, leveraging the strengths of DeepInsight and augmenting it with quality control and batch normalization measures, to cater specifically to the intricacies of single-cell analysis. Figure 6 presents an overview of the scDeepInsight on the left-hand side. On the right-hand side, the figure contrasts the true cell annotation with the cell annotation predicted by scDeepInsight.

scDeepInsight revolutionizes the cell type annotation by applying the tabular-to-image transformation methodology to scRNA-seq data. This transformed representation, combined with the analytical strength of CNNs, facilitates precise and robust cell-type identification. Instead of relying solely on known markers, the model harnesses the whole transcriptomic profile of individual cells, offering a more comprehensive classification. This is done by training scDeepInsight on reference dataset and identifying cell types for query datasets. The performance improvement noted was over 7% compared to other competing methods.

### Discovering new cell types

One of the most promising facets of scDeepInsight is its potential to unearth previously undiscovered or rare cell types. By transforming the scRNA-seq data into a visual landscape, clusters that might be overlooked or merged in traditional analyses stand out. These unique clusters represent potential new cell types or transitional states, furthering our understanding of tissue biology, developmental processes, and disease mechanisms.

### Identifying marker genes

Marker genes play a critical role in cell-type identification, offering biological insights into the function and nature of various cell populations. Beyond classification, scDeepInsight also aids in the identification of these marker genes. By reverse engineering the image-like representation and linking it back to genomics data, one can pinpoint genes that are distinctly expressed in specific cell types. This not only solidifies the identification but also provides a foundation for functional assays, therapeutic targeting, and further biological inquiries.

In essence, scDeepInsight amplifies the power of single-cell RNA sequencing, providing tools not just for identification but also discovery. As the world of genomics moves towards higher resolution, tools like scDeepInsight will be instrumental in ensuring that we fully harness the data’s potential, advancing both science and medicine.

## Conclusions and future perspectives

As the boundaries of genomics continue to expand, our analytical strategies must evolve in tandem. DeepInsight and its derivatives represent a monumental leap in this progression. They merge the worlds of image-based data analysis with omic data, facilitating nuanced interpretations that were previously challenging to achieve:

1. Redefining omic data interpretation: The transformation of omic data into image-like representations through DeepInsight or similar technology has undoubtedly expanded our analytical capacities and robustness by capturing latent structures and coherences behind data, e.g. omics data. Its adaptability, as seen in its fusion with diverse methodologies highlights the technique’s dynamic potential.
2. Holistic multi-omic integration: The emergence of integrative models highlights the growing need for more comprehensive approaches within genomics. As reliance on single omic data might become limiting, the future could see a heightened dependence on tools like DeepInsight and its derivatives, e.g. DeepInsight-3D, to provide a holistic perspective on biological systems. Their applications, especially in fields such as anti-cancer drug response prediction, underscore their potential clinical relevance.
3. Decoding cellular heterogeneity: Single-cell analyses, powered by tools like scDeepInsight, have transformed our understanding of cellular landscapes. The discovery of new cell types and marker genes underscores its potential to contribute immensely to cell biology.
  While the amalgamation of tabular-to-image conversion with CNNs for omics analysis has propelled significant advancements, still there exists a panorama of challenges that require resolution:
4. Interpretability and biological relevance: The “black box” nature of DL models, including CNNs, has led to the emergence of techniques like CAMs and DeepFeature. While such tools offer promise, ensuring interpretability and preserving the biological relevance of data representations are paramount challenges.
5. Data challenges, model complexity, and overfitting: Omics data is intrinsically heterogeneous, encompassing information like genomic, epigenomic, transcriptomic, proteomic, and metabolomic data. Adapting these diverse data types and handling issues of data scaling, size, and overfitting are substantial concerns. Striking a balance between model complexity, capacity, and available data is essential to prevent overfitting, especially when dealing with high-dimensional omics data.
6. Technical challenges: Issues like hyperparameter tuning, computational resource limitations, and model generalizability across different conditions and platforms need addressing.
7. Integration and benchmarking: Infusing domain-specific knowledge into model training and rigorous benchmarking against established methods is crucial for assessing the true potential of a model.
8. Future horizons: The confluence of DL and biology, as manifested by these methodologies, will likely intensify in the coming years. We can envision a future where real-time omic data transformation and analysis become standard in clinical settings, expediting diagnostic and therapeutic decisions. Moreover, the emergence of even more robust models, adaptable to a diverse range of omic data types, is anticipated.
9. Towards personalized medicine: The culmination of these advancements aims to customize medical interventions to individuals. Harnessing insights from tabular-to-image converters and CNNs trained on vast datasets, we approach the realization of truly personalized medicine. Whether it pertains to drug responses, uncovering molecular mechanisms, or identifying novel cellular states, these tools hold promise in crafting treatments attuned to an individual’s genetic blueprint.

Challenges, such as those listed above, underline the importance of interdisciplinary collaboration between ML experts, bioinformatics professionals, biologists, medical researchers and doctors, and patients. Such collaborations will be pivotal in advancing the DL models, expanding the horizons of omics data analysis.

In conclusion, as we stand on the cusp of this analytical revolution in genomics, it is imperative to embrace these novel methodologies. Their potential to revolutionize our comprehension of biology, combined with profound clinical implications, cements their role as indispensable instruments in our endeavor to decode the intricacies of life.
